# Non-genetic health professionals’ attitude towards, knowledge of and skills in discussing and ordering genetic testing for hereditary cancer

**DOI:** 10.1007/s10689-015-9852-6

**Published:** 2015-11-21

**Authors:** Kirsten F. L. Douma, Ellen M. A. Smets, Dawn C. Allain

**Affiliations:** Department of Medical Psychology, Academic Medical Center/University of Amsterdam, P.O. Box 22660, 1100 DD Amsterdam, The Netherlands; Division of Human Genetics, The Ohio State University Wexner Medical Center, Columbus, OH USA

**Keywords:** Communication skills, Cancer genetics, Doctor–patient communication, Knowledge

## Abstract

Non-genetic health professionals (NGHPs) have insufficient knowledge of cancer genetics, express educational needs and are unprepared to counsel their patients regarding their genetic test results. So far, it is unclear how NGHPs perceive *their* own communication skills. This study was undertaken to gain insight in their perceptions, attitudes and knowledge. Two publically accessible databases were used to invite NGHPs providing cancer genetic services to complete a questionnaire. The survey assessed: sociodemographic attributes, experience in ordering hereditary cancer genetic testing, attitude, knowledge, perception of communication skills (e.g. information giving, decision-making) and educational needs. Of all respondents (N = 49, response rate 11 %), most have a positive view of their own information giving (mean = 53.91, range 13–65) and decision making skills (64–77 % depending on topic). NGHPs feel responsible for enabling disease and treatment related behavior (89–91 %). However, 20–30 % reported difficulties managing patients’ emotions and did not see management of long-term emotions as their responsibility. Correct answers on knowledge questions ranged between 41 and 96 %. Higher knowledge was associated with more confidence in NGHPs’ own communication skills (r_s_ = .33, *p* = 0.03). Although NGHPs have a positive view of their communication skills, they perceive more difficulties managing emotions. The association between less confidence in communication skills and lower knowledge level suggests awareness of knowledge gaps affects confidence. NGHPs might benefit from education about managing client emotions. Further research using observation of actual counselling consultations is needed to investigate the skills of this specific group of providers.

## Introduction

Since the early 1990s the possibilities for DNA testing in cancer have rapidly expanded. Through the combination of pedigree risk assessment and genetic testing it can be determined whether a patient’s personal or family history of cancer has an underlying hereditary cause. Genetic information about cancer is complex and involves understanding risks and inheritance patterns. Many individuals find such complex information difficult to understand [1, 2]. Explaining genetic information to patients or at-risk family members is demanding and has traditionally been carried out by trained medical geneticists and genetic counselors. Research shows that clinical geneticists and genetic counselors in general are well trained to provide genetic information [3]. Hence, they have an important role in providing the information in an understandable way.

Over the last decade, though, more genetic tests, especially in the United States, are being ordered by non-genetic health professionals, such as oncologists, gynecologists, and primary care providers [4, 5]. The American Society of Clinical Oncology stressed that the burden on oncologists becomes greater in fully explaining issues surrounding cancer risks, including genetic risks. Also, they need to be prepared to order genetic tests themselves and to be responsible for appropriate follow-up care. Therefore, they need to know of recent genetic advances [6, 7]. This level of preparedness is not only necessary for oncologists, but also for other health professionals caring for cancer patients.

The six-function medical communication model of de Haes and Bensing [8] states that medical communication should include (1) fostering the relationship, (2) gathering information, (3) information provision, (4) decision making, (5) enabling disease and treatment-related behavior, and (6) responding to emotions. Communication about cancer genetics requires all of these communication tasks. First, proper gathering of information is necessary to identify someone at high risk. Second, information provision must be adequate to inform patients and family members not only with basic information about heredity, but also about the most adequate treatment and/or preventive measures. Third, ordering a genetic test asks for adequate information exchange and (shared) decision-making. Fourth, motivational communication to enable patients to follow screening recommendations and undergo prophylactic surgery, when necessary, is an important task. Within all communication about genetic testing for cancer, emotions with regard to the test result and consequences for the patient and their family should be addressed.

Numerous studies have shown that non-genetic health professionals have insufficient knowledge of genetics, express educational needs and are generally unprepared to counsel their patients regarding genetic test results [9–14]. Also, family history taking [15, 16] and risk communication [17] are perceived as difficult tasks by non-geneticists. Inadequate genetic counseling and testing can lead to negative outcomes in patients and their families [18, 19]. However, not much is known about the attitude towards, knowledge of and communication skills in discussing and ordering genetic testing of non-genetic health professionals (NGHPs) who order genetic tests *themselves*.

The current study was undertaken to gain insight into the attitude, knowledge and skills of NGHPs who provide genetic testing. Investigating the perceptions of non-genetic health professionals about the communication process will provide insight in their perceived barriers and challenges to genetic counseling and testing. If deemed necessary this could aid in the development of an intervention to support and enhance their communication skills.

We also wondered if NGHPs are aware of their knowledge level. A study by Klitzman et al. [10] showed that internists who rated (subjectively) their knowledge as poor, and were uncomfortable ordering testing and counseling patients, still ordered genetic tests. Therefore, in this study we investigate if NGHPs’ objective knowledge level is associated with their self-perceived information giving skills. We hypothesize that if there is an association this might point to awareness of limitations and thus more willingness to receive further education.

The primary research questions are:

1. How do NGHPs providing genetic testing perceive their own communication behavior (attitude, knowledge, skills) in cancer gene testing?
2. What education and/or training have NGHPs received with regard to communication about cancer gene testing and what are their needs in this regard?
3. What is the level of knowledge NGHPs have about cancer gene testing and is it associated with self-perceived information giving skills?

## Materials and methods

### Study sample and procedures

The Myriad Genetics Find a Healthcare Provider website (www.myriadtests.com/finddoc.php) and the National Cancer Institute (NCI) Cancer Genetics Services Directory (www.cancer/gov/cancertopics/genetics/directory) are publically accessible databases containing contact information for healthcare providers who have self-identified as cancer genetic service providers. At the time of this study, Myriad Genetic Laboratories was the sole provider of genetic testing for the *BRCA1* and *BRCA2* genes in the United States. The Find A Provider section on their website was a mechanism for patients to identify healthcare professionals in their communities who provided genetic testing. Listing on the website was completely voluntary and the list was managed by Myriad Genetic Laboratories. The database of the NCI lists professionals who provide services related to cancer genetics (cancer risk assessment, genetic counseling, genetic susceptibility testing, and others). Professionals must apply to be listed in this directory and must meet certain criteria. Inclusion in the directory does not imply an endorsement by the National Cancer Institute. Professionals listed are contacted yearly by the NCI through e-mail to verify and/or update their record information.

For this study, we utilized these databases, to identify our study population. Study eligibility was as follows: (1) healthcare professionals self-identified as providing cancer genetic services with contact information listed on the Myriad Genetics Find a Healthcare Provider or NCI Cancer Genetics Services Directory and (2) providers of care in one of four Midwest states (Ohio, Michigan, Indiana, and Kentucky). Individuals who met the eligibility criteria were sent a letter of invitation explaining the aim of the study along with a paper version of the survey and a return envelope. The letter of invitation also included a link to an online version of the survey. A reminder was sent after 2 weeks. Completion and return of the study questionnaire implied consent. Health professionals who completed the survey and reported they were trained as master-degree genetic counselors, advanced practice nurses in genetics, or medical geneticists were excluded from data analysis.

Because of a low response rate we expanded the study to four more states (Texas, California, New York, and Massachusetts). The procedure was the same. However, this time a prize draw for two Amazon gift cards of fifty dollars and the possibility to receive a report about the results of the study were added, trying to increase the response rate.

The IRB of The Ohio State University approved the study.

### Measures

#### Questionnaire

Questionnaire items assessed: (1) Sociodemographic characteristics, (2) Practice characteristics, (3) Information giving, (4) Decision-making, (5) Communication about disease and treatment related behaviors, (6) Managing emotions, (7) Education and (8) Knowledge. Table 1 shows the details of the measures used.

**Table 1:** Measures

Topic	Subcategory	Number of items	Scoring	Alpha	Reference	Description of questions/examples
1. Sociodemographic characteristics	n/a	n/a	n/a	n/a	n/a	Age, gender, race, specialty
2. Practice characteristics	n/a	n/a	n/a	n/a	n/a	specialty, number and type of gene tests ordered, experience with patient care
3. Information giving	a. Topics discussed	5	5-point scale: always to never	n/a	5 self-developed statements on how often topics are discussed when providing cancer genetic counselling	e.g. ‘Benefits and limitations of close cancer surveillance’
	b. Skills perception	13	13–65 (5-point scale: strongly agree to strongly disagree). A higher total score means a more positive perception of one’s info giving skills	0.88	10 items are adapted from an unpublished questionnaire used in the radiotherapy setting and 3 items are based on Keating et al. [4]	e.g. ‘I am comfortable discussing hereditary cancer issues with my patients’
4. Decision-making	a. Attitude towards patient autonomy	14		See Table 3	Adaptation of the Ideals of Patient Autonomy Scale (Stiggelbout et al 2012) [30]. Our adapted version includes 3 subscales (see Table 3)	e.g. ‘If the patient does not want to receive information about risks, the healthcare provider should respect this’
	b. Attitude towards responsibility	1	5-point scale (strongly agree to strongly disagree)		Self-developed. This item is added to the adapted IPAS in Table 3	‘It is my responsibility to help a patient make a decision about genetic testing’
	c. Skills perception	6	5-point scale (difficult to easy)		Self-developed	Rate the difficulty of several communication tasks related to decision-making, e.g. ‘involving the patient in the decision’
5. Enabling disease and treatment related behaviors	a. Attitude towards responsibility	2	5-point scale (strongly agree to strongly disagree)		Self-developed	e.g. ‘It is my responsibility to discuss preventive behaviors such as prophylactic surgeries and/or regular cancer screening’
	b. Attitude towards future developments	2	5-point scale (strongly agree to strongly disagree)		Self-developed and one item based on Shields et al. [5]	e.g. ‘I am optimistic that genetic research will lead to significant improvements in the treatment of complex traits’
6. Managing emotions	a. Skills perception	7	5-point scale (difficult to easy)		Self-developed	Rate the difficulty of several communication tasks related to managing emotions, e.g. ‘preparing the patient for negative emotions’
	b. Attitude	2	5-point scale (strongly agree to strongly disagree)		Self-developed	e.g. ‘It is my responsibility to manage emotions that patients experience during genetic counseling’
7. Education	a. Received training	7	2 Yes/No, 2 multiple-options, 2 open questions, 1 statement with 5-point scale (strongly agree to strongly disagree)		Questions based on Shield et al. [5] and on remarks in Zon et al. [7]	e.g. ‘Did you receive specific training about how to communicate with patients about hereditary cancer?’
	b. Use of risk models	3	2 Yes/No, 1 multiple-options		Questions based on remarks in Zon et al. [7]	e.g. ‘Which web-based risk assessment models do you use?’
8. Knowledge	a. General perception	4	4-point scale (Very good to very poor)		Based on Klitzman et al. [10]	e.g. ‘My knowledge about hereditary cancer genetics is…’
	b. Confidence	2	5-point scale (Strongly agree to strongly disagree)		Based on Shields et al. [5]	e.g. ‘I am confident in my ability to interpret a variant genetic test result’
	c. Objective knowledge	9	3-point scale (True/False/Do not know)		Several items are derived from Erblich et al. [31] and 3 items are derived from a study among FAP-patients of one of the authors (Douma)	

### Data analysis

Descriptive statistics were used to describe the study sample. Sum scores were calculated for Information giving Skills perception (see 3b in Table 1) and Objective knowledge (see 8c in Table 1). The total knowledge score was calculated by assigning one point for every correct answer (range 0–9). A mean correct knowledge score was calculated as the mean number of correct answers on the knowledge items. In addition, the percentage of correct answers for each item was analyzed.

We calculated reliability for the three subscales of the adapted Ideal Patient Autonomy Scale (IPAS) (see Table 1). Because alpha’s are low, results for individual items are presented. Pearson’ correlation was calculated for the association between the total score of Perception of Information giving skills and the Knowledge score. All analyses were carried out using IBM SPSS version 20.0. A *p* value of .05 (two-sided) was considered significant.

## Results

### Sample characteristics

In the first mailing (summer 2013) 151 invitations were sent of which 17 were not delivered due to incorrect address (returned to sender). In the second mailing (April 2014), we sent out 366 invitations of which 70 returned for incorrect address. In total, we received 49 questionnaires (11 % response rate). One filled-in questionnaire was removed because of many missing values, four others were removed because they were filled in by participants specialized in genetic counseling or medical genetics. Table 2 displays the characteristics of the sample.

**Table 2:** Sample characteristics (n = 44)

Variable	N	%
Age		
25–34	2	5
35–44	10	23
45–54	12	27
55–64	15	34
65–74	4	9
75 or older	1	2
Gender		
Male	19	43
Female	25	57
Race		
Caucasian/white	43	98
Other	1	2
Specialty		
Gynecology/obstetrics	20	46
Surgical oncology	10	23
Medical oncology	6	14
Family medicine	2	5
Gastroenterology	1	2
Other	5	11
Number of gene tests ordered for inherited cancer susceptibility in past year^a^		
1–10	7	16
11–20	8	19
21–30	8	19
31–40	3	7
41–50	2	5
51 or more	15	35
Ordered testing for^b^		
Breast and ovarian cancer	42	100
Colorectal cancer	34	81
Endometrial cancer	25	60
Melanoma	16	39
Pancreatic cancer	16	39
Other	5	12
Years of experience in patient care		
1–9	4	9
10–19	14	32
20–29	13	30
30 or more	13	30

^a^One missing value

^b^Missing values are not included in the calculation of percentages. Three persons had missing values on this question

### Information giving

NGHPs reported addressing most relevant topics when providing cancer genetic counseling. These topics included: benefits and limitations of close cancer surveillance (95 % always/frequently discuss this), sharing test results with family members (91 %), benefits and limitations of prophylactic surgery (89 %) and confidentiality and privacy (89 %). The possibility of a negative psychological reaction to genetic testing was least often discussed (73 vs. 89–95 %).

NGHPs positively perceived their own information giving skills with a mean score 53.91 (SD = 6.8) on a scale of 13–65. Individual items showed that NGHPs found it most difficult to inform a patient about a variant test result: 16 % (strongly) agreed, while 23 % neither agreed or disagreed. Also, informing minority patients [14 % (strongly) agreed, 16 % neither agreed nor disagreed] and lower educated patients [14 % (strongly) agreed, 27 % neither agreed nor disagreed] was perceived as difficult by some. In addition, 14 % found it difficult to ensure that patients understand the genetic test result (27 % neither agreed nor disagreed) and 14 % found it difficult to inform a patient about a positive test result (11 % neither agreed nor disagreed).

### Decision-making

On average, NHGPs’ opinion was that patients know best regarding decisions about genetic testing [mean score is 3.92 (SD = 0.50)] and patients should decide [mean score is 1.69 (SD = 0.59)]. Furthermore, opinions diverged as to patients’ right not to pursue genetic testing [mean score is 2.77 (SD = 0.80)]. Table 3 shows the responses to the individual items of the adapted IPAS.

**Table 3:** Adapted Ideal Patient Autonomy Scale

Item (with original item numbers)	(Strongly) Agree		Neither agree or disagree		(Strongly) Disagree	
	n	%	n	%	n	%
Scale: Doctors knows best (α = 0.61)						
2. It is better that the healthcare provider rather than the patient decides about genetic testing	0	0	3	7	41	93
5. During the conversation, the patient must entrust him/herself to the expertise of the healthcare provider	24	55	13	30	7	16
9. If the healthcare provider and the patient cannot agree on whether or not to undergo genetic testing, the healthcare provider should make the final decision	0	0	3	7	41	93
10. The patient should, without much information on the consequences, confidently undergo genetic testing	2	5	4	9	38	86
12. The healthcare provider can presume that the patient knows the consequences of receiving a genetic test result	1	2	6	14	37	84
Scale: Patient should decide (α = 0.45)						
6. The patient must choose between whether to undergo genetic testing or not	39	89	1	2	4	9
11. It would be taking things too far when the healthcare provider would decide for the patient	38	86	3	7	3	7
14. As it concerns the body and life of the patient, the patient should make decisions about genetic testing	41	93	2	5	1	2
Scale: Right to non-participation (α = 0.42)						
4. Patients should have the right not to be involved in the decision about genetic testing	13	30	7	16	24	55
8. Patients who become afraid when deciding about genetic testing should be left in peace by the healthcare provider	14	32	14	32	16	36
13. If a patient chooses not to know anything about their genetic risk, the healthcare provider should respect this	37	84	2	5	5	11
Items not included in a subscale						
1. It is my responsibility to help a patient make a decision about genetic testing^a^	30	68	7	16	7	16
3. If a healthcare provider and patient properly consult with each other, it does not matter who makes the final decision about genetic testing	9	20	3	7	32	73
7. Before a patient consents to genetic testing she/he should receive all information on the consequences of the test result	41	93	1	2	2	5

^a^Item 1 is self-developed (see Table 1)

**Fig. 1:**
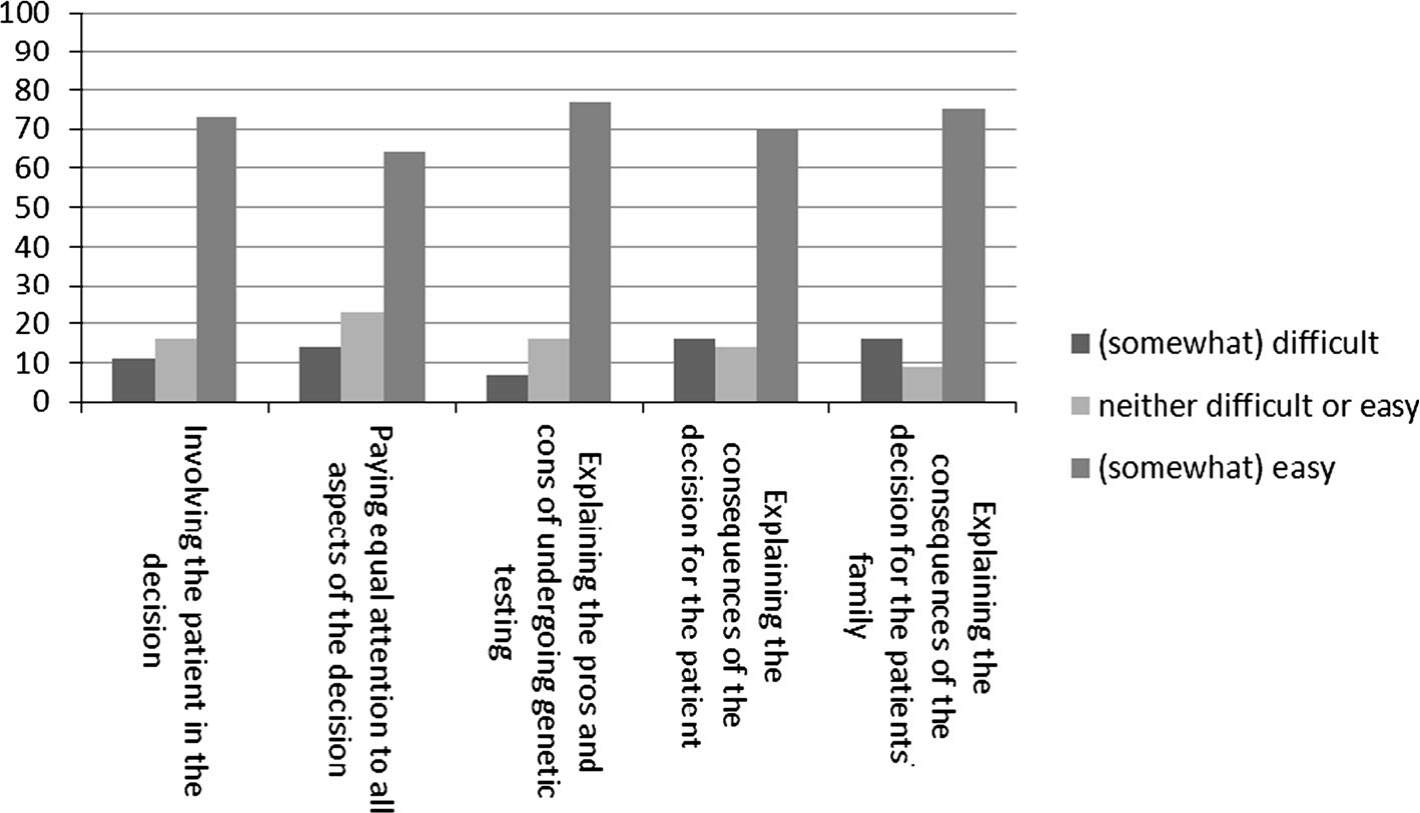
Decision-making communication tasks

Figure 1 shows that 7–16 % of NHGPs perceived communication tasks related to decision-making as difficult, depending on the task. They experienced most difficulties explaining the consequences of genetic testing for the patient as well as the family members.

### Enabling disease- and treatment related behavior

Ninety-eight percent (n = 43) of NHGPs agreed that it was their responsibility to discuss preventive behaviors with their patients, while only 2 % (n = 1) disagreed. Furthermore, 91 % (n = 40) agreed that it was their responsibility to remind patients about their screening, 2 % (n = 1) neither agreed or disagreed, while 7 % (n = 3) disagreed.

Ninety-five percent (n = 42) of NHGPs were optimistic that genetic research will lead to significant improvements in the treatment of complex traits, while 5 % (n = 2) neither agreed or disagreed. Seventy-seven percent (n = 34) agreed that personalized risk information will motivate people to change their behavior, while 18 % (n = 8) neither agreed or disagreed and 5 % (n = 2) disagreed.

### Managing emotions

**Fig. 2:**
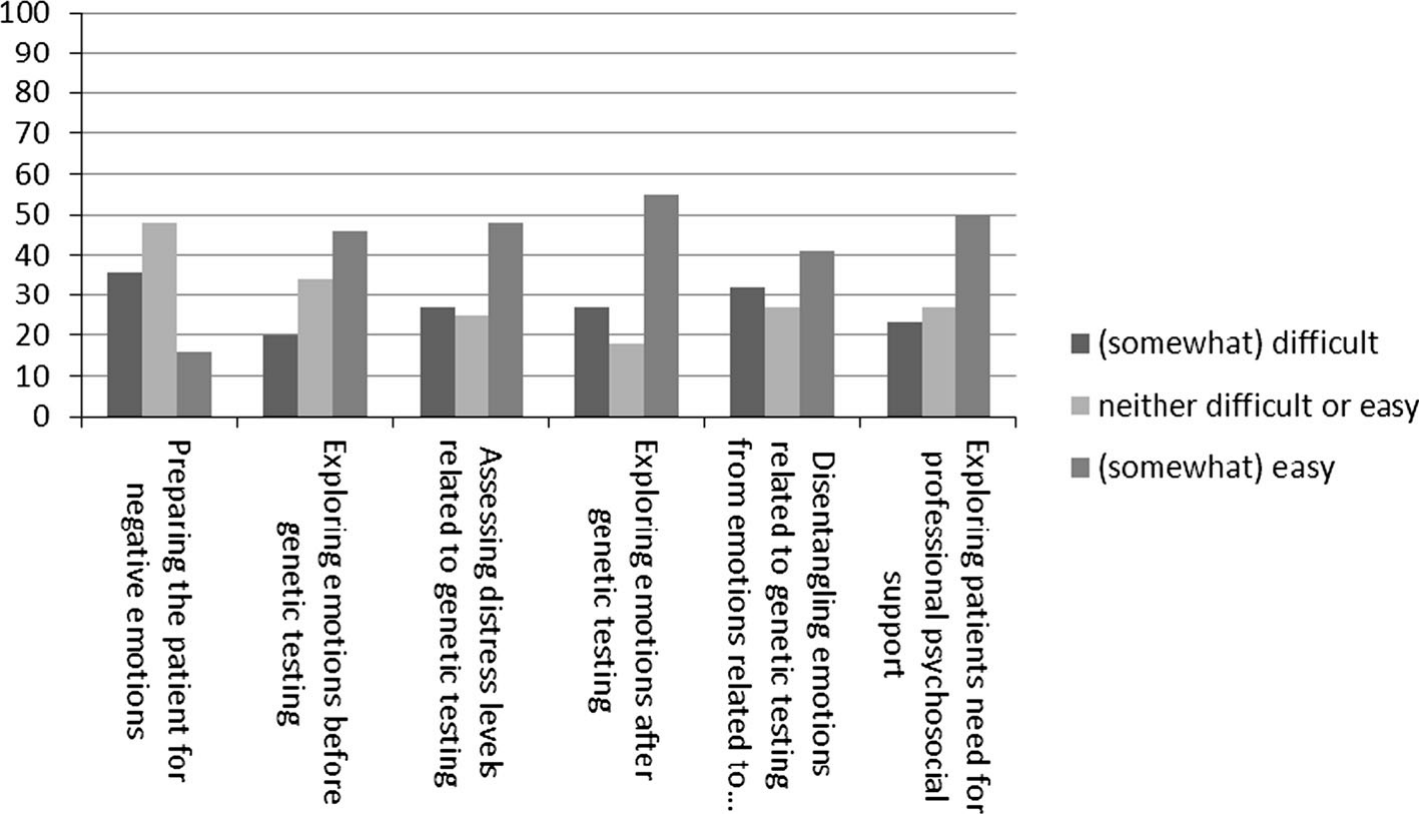
Managing emotions

Figure 2 shows that 20–36 % of NHGPs perceived communication tasks related to managing emotions as difficult, depending on the task. Especially, preparing the patient for negative emotions and disentangling of emotions related to genetic testing from emotions related to the disease itself were perceived as difficult.

Seventy-one percent (n = 31) saw it as their responsibility to the manage emotions patients experience during genetic counseling, while 18 % (n = 8) neither agreed nor disagreed and 11 % (n = 5) disagreed with this. Thirty-nine percent (n = 17) saw it as their responsibility to manage the long-term emotions (>3 months) patients experience after a genetic test, while 30 % (n = 13) neither agreed or disagreed and 32 % (n = 14) disagreed.

### Education

Fifty-five percent (n = 24) of respondents received specific training about how to communicate with patients about hereditary cancer. This training was received either through continuing medical education (CME) (36 %), a Myriad-sponsored course (29 %), fellowship training (9 %), medical school or residency (9 %) or others (such as an intensive courses on genetics; 15 %).

Participants reported to educate themselves about the most recent advances in genetic testing with CME (91 %; n = 40), journal articles (84 %; n = 37), on genetic laboratory sponsored trainings (55 %; n = 24), through colleagues (46 %; n = 20) and on the job training (41 %, n = 18).

Ninety-one percent (n = 40) felt confident about taking a cancer genetic family history [(strongly) agree], while 9 % (n = 4) neither agreed or disagreed. Thirty-six percent (n = 16) used web-based tools for taking a family history. Main reasons for not using these tools are time (n = 6), unfamiliarity (n = 5), use a paper form (n = 5), prefer orally discussing family history (n = 3) and other (n = 8). Seventy-four percent (n = 31) used web-based risk assessment models. Of those not using web-based risk assessment tools 79 % (n = 11) would be interested to use them.

### Knowledge

**Fig. 3:**
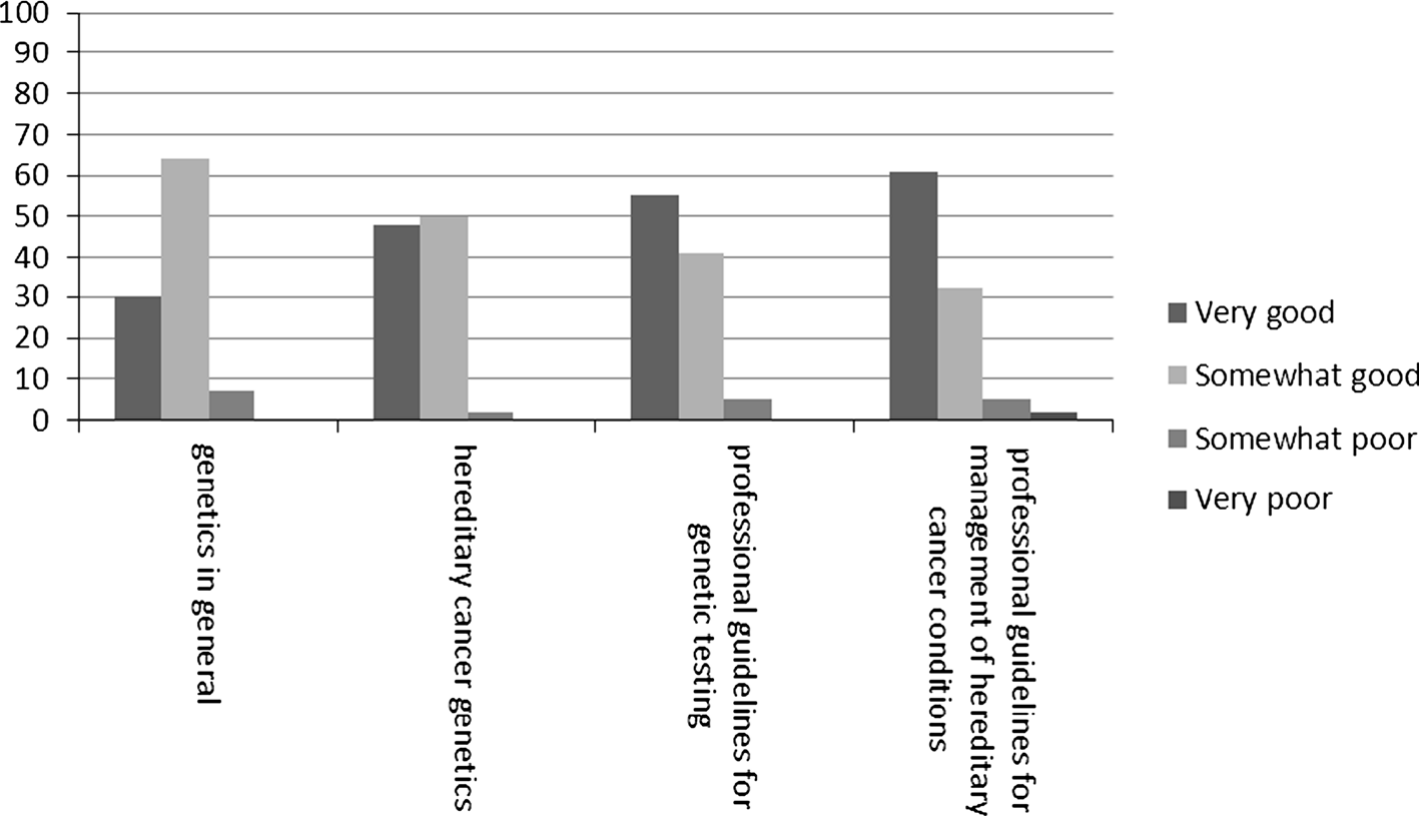
Perception of own oncogenetic knowledge

Figure 3 shows that 30–61 % of NHGPs judged their knowledge on genetics as very good, while 32–64 % judged it as somewhat good, depending on the specific topic.

Sixty-eight percent (n = 30) agreed or strongly agreed that they were confident about their ability to interpret a variant cancer genetic test result, while 23 % (n = 10) neither agreed or disagreed and 9 % (n = 4) disagreed. Ninety-eight percent (n = 43) agreed or strongly agreed that they are confident about their ability to interpret a negative cancer genetic test result, while 2 % (n = 1) neither agreed or disagreed.

The mean knowledge score was 6.5 (SD = 1.7). Table 4 shows that the percentage of participants giving the correct answer ranged between 41 and 96 %. Only three persons gave the correct answer to all knowledge questions.

**Table 4:** Participants’ knowledge about hereditary cancer

Statement	Participants who gave correct answer (%)
If a women’s BRCA1 or BRCA2 gene result shows a variant of unknown significance, other affected family members need to be tested in order to determine the meaning of the result. (false)	41
If a woman’s BRCA1 or BRCA2 gene result reveals a positive test, she should be counseled to have her ovaries surgically removed after she is done having children. (true)	96
If a father has a mutation in the APC gene (Familial adenomatous polyposis (FAP), his children have a 50 % chance (1 in 2) for carrying this mutation as well. (true)	80
After removal of colon polyps for an FAP diagnosis regular bowel examinations are no longer necessary. (false)	91
A hereditary predisposition to FAP can skip a generation. (false)	52
If a person has colorectal cancer at age 49 and also has a family member with endometrial cancer diagnosed at age 60 years, genetic testing is indicated. (true)	84
A person with uterine cancer at 49 years of age has an indication for genetic counseling. (true)	57
A person with two melanomas has an indication for genetic counseling. (true)	72
If a female is found to have a BRCA mutation and her sister’s BRCA result is negative, the sister is still at increased risk for developing ovarian cancer. (false)	68

There was a significant positive correlation between Objective knowledge and Information giving Skills perception, r_s_ = .33, *p* = 0.03, meaning that participants with more knowledge had a more positive view on their own information giving skills.

## Discussion

In contrast to known studies showing that providers are unprepared to counsel their patients [9–14], our study shows that most non-genetic health professionals ordering cancer gene testing have a positive attitude towards, knowledge of and skills in discussing and ordering genetic testing for cancer.

To be able to interpret these findings we first need to address the low response rate (11 %) of our study. Our study may have suffered from response bias, as our respondents may be those who view this as an important process whereas those who do not may not have responded. Also, our data shows that respondents were individuals with a lot of experience in ordering cancer gene testing (47 % ordering more than 30 gene tests a year) and half of them received training regarding communication with patients about hereditary cancer. These findings suggest an overrepresentation of experienced and well-trained non-genetic health professionals. If the low response rate reflects a lack of interest, this is worrisome. Those who may not be as comfortable with communicating informed consent may not be answering and thus it is hard to address what they specifically need for resources. Furthermore, while all individuals in the databases identified themselves as ordering genetic testing we received many returned envelopes, suggesting that these databases were not as up to date or representative as may be suggested.

So, how to interpret the positive view of NGHPs of discussing and ordering genetic tests for cancer? Especially, as the wealth of available research literature suggest that non-genetic health professionals experience difficulties in genetic counseling [9, 11, 18, 20–22]. Of note, medical specialists and primary care physicians (as in many of the other studies cited) are quite different from our NGHPs who are part of a database of providers identifying themselves as providers of genetic testing. Our respondents, with a high interest in genetic testing, might indeed have better communication skills or felt the need, because of social desirability, to at least give this impression. Future research might use observations of genetic counseling by non-genetic health professionals ordering their own genetic testing to investigate the skills of this specific group.

Our knowledge level seems to be higher than observed in most other studies where correct answers to knowledge questions regarding cancer gene testing in non-genetic health professionals ranged between 13 and 48 % [13, 23–25] ). In contrast, we also found a study among medical specialists showing higher levels of knowledge ranging between 72 and 100 % [26]. It is however hard to compare these studies as knowledge questions are quite different in each one of them and different health professionals are included. It is important to note however, that the high degree of variability in knowledge scores could raise concern regarding accuracy of information being given to some patients. Only three respondents in our study were able to answer all knowledge questions correctly. Investigation of individual items shows that respondents know least about consequences for other family members. This might lead to family members being less well informed if people get tested through a NGHP. This definitely warrants further investigation. Also, the fact that more than 30 % of participants are unsure how to interpret a variance of unknown significance (VUS) is a reason for concern, as the number of VUS will increase with the current application of next generation sequencing (NGS) in the United States.

Although most NGHPs in our study have an overall positive view, they report more difficulties managing emotions. In addition, they do not perceive management of long-term emotions as their responsibility. The majority of our respondents however are involved in long-term management of at-risk patients and should be prepared to address these emotions.

Slightly more than half of our respondents have received education regarding communication about genetic testing. The low percentage of NGHPs who received training is a reason for concern. In the last decennium *genetic* health professionals have moved, at least theoretically, from non-directive counseling to counseling based on principles of shared decision making [27–29]. However, NGHPs in our study still seem to have a preference for non-directive counseling in which patients decides. NGHPs may not be well informed about the benefits of shared decision making in genetic counseling.

Our study showed that the higher the knowledge level, the more confidence non-genetic health professionals reported in information giving skills. This association suggests that awareness of knowledge gaps affects health professionals’ confidence. Would identifying knowledge gaps in individual NGHPs be a helpful route towards enhancing quality and consistency of care among providers performing genetic risk assessment, counselling and testing? Interestingly, Prochniaks’ study suggests that confidence plays an important role in preference of physicians towards ordering genetic testing themselves or rather refer to a clinical genetic center [11]. In their study individuals who had the highest knowledge level were significantly more likely to have a preference for referral to a cancer genetics center instead of counseling the patient themselves. This suggests that knowing your limitations makes NGHPs more willing to get further education. In addition, this could also mean that they value more the contribution that genetic professionals make to the emotional aspects that arise during counseling sessions and the genetic professional expertise.

Overall, NGHPs who participated in our study have a positive view on their attitude towards, knowledge of and skills in discussing and ordering genetic testing. However, our study raises several concerns about how well-informed patients and their families will be. Specific attention is needed for the consequences of genetic testing for family members and the interpretation of VUS. Continuing medical education should address these issues.
